# Associating cancer-related RNA structure disrupting SNPs in LincRNAs to function

**DOI:** 10.1186/s12864-025-12226-0

**Published:** 2025-12-12

**Authors:** Xueer Han, Christian Anthon, Adrian Sven Geissler, Radhakrishnan Sabarinathan, Stefan Ernst Seemann, Jakob Hull Havgaard, Jan Gorodkin

**Affiliations:** 1 Department of Veterinary and Animal Sciences, Center for non-coding RNA in Technology and Health, University of Copenhagen, Copenhagen, Denmark; 2 National Centre for Biological Sciences, Tata Institute of Fundamental Research, Bengaluru, Karnataka India

**Keywords:** Long non-coding RNA, Single nucleotide polymorphism, RNA secondary structure, Human cancer, Structural disruption, RNA binding protein, RNA methylation, Long intergenic non-coding RNA

## Abstract

**Background:**

Long intergenic non-coding RNAs (lincRNAs) and single nucleotide polymorphisms (SNPs) have been associated with cancers for years, yet the molecular mechanism is mostly unclear. The secondary structure of lincRNAs is often crucial for their biological function but can be disrupted by SNPs. However, only a small number of studies investigated how lincRNAs function through secondary structure. Given that the vast majority of cancer-related SNPs are located mainly in non-coding regions, there is a large potential for associating SNPs to disrupted structure in lincRNAs.

**Methods:**

To estimate the structural impacts of cancer-associated SNPs on lincRNAs, we predicted local secondary structures for lincRNAs and computed structural distances between structural ensembles of the wild-type and mutant sequences. Manual literature curation was performed to study the function of lincRNAs that are structurally disrupted by cancer-associated SNPs. By integrating with RBP binding sites annotation, we estimated the impacts of structural changes from cancer-associated SNPs on the protein binding of lincRNAs.

**Results:**

We predict 559 SNPs to cause significant structural disruption in 231 lincRNAs using RNAsnp (*P*-value < 0.1). In addition, we find that these disrupted regions have the potential to alter the binding ability of lincRNAs with RBPs. An example is the structural change in the lincRNA small nucleolar RNA host gene 25 (SNHG25), which overlaps the binding site of protein insulin like growth factor 2 mRNA binding protein 2 (IGF2BP2) in glioblastoma multiforme.

**Conclusions:**

The results show the importance of the lincRNA secondary structure in understanding their biological function, especially the structural changes from SNPs in cancer. The predicted structural change in lincRNA SNHG25 holds a potential insight into the mechanism of protein IGF2BP2 in recognizing RNA methylation signals in glioblastoma multiforme.

**Supplementary Information:**

The online version contains supplementary material available at 10.1186/s12864-025-12226-0.

## Background

Single nucleotide polymorphisms (SNPs) or single nucleotide variants are the most common human DNA variants and exist widely in the human genome [1]. SNPs are related to many complex diseases (e.g., various cancers), and the mechanisms rely on the genetic region where the SNPs are located [2]. They have been reported to be associated with both overall cancer risk [3] and susceptibility to some specific cancers, such as breast cancer [4] and prostate cancer [5]. Specifically, the majority of cancer-associated variants are found in non-protein-coding regions [6], indicating the importance of functional changes induced by SNPs in the non-protein-coding regions in understanding cancer.

Long intergenic non-coding RNAs (lincRNAs) are a subtype of long non-protein-coding RNAs (lncRNAs) that do not overlap with protein-coding genes [7]. Studies on lincRNAs have a long history due to no overlap with protein-coding loci and consequently distinguish their function from protein-coding gene [8]. Here, we also focus on lincRNAs because SNPs in lncRNAs overlapping with protein-coding genes can impact both lncRNA and protein functions. LincRNAs have been widely reported to be differentially expressed in cancers [9]. The functions of lincRNAs are performed through binding to other molecules [10], either through sequence complementarity (e.g., with DNA and miRNA) or specific RNA structure recognition (e.g., with protein). In this study we focus on the function of lincRNAs through their binding to proteins.

Like other molecules, lincRNA structure plays a key role in performing the biological function and can be disrupted by mutations. A single nucleotide change can disrupt, alter or induce a (secondary) structure of an RNA molecule. This is equivalent to how SNPs can change the structure in proteins and result in change in function. Whereas there is not a term for this as for proteins, this has been referred to as “RiboSNitch” for RNAs [11]. For example, two mutations together, comprised of three nucleotide changes in motif 1 and four nucleotide changes in motif 2 of lincRNA steroid receptor RNA activator-like non-coding RNA 1 (SLNCR1), altered the secondary structure of SLNCR1 [12], as confirmed by changes in chemical reactivity detected in Selective 2′-Hydroxyl Acylation analyzed by Primer Extension and Mutational Profiling (SHAPE-MaP) experiments [13]. SHAPE-MaP is a popular experimental method to analyze RNA secondary structure by chemically probing RNA flexibility and encoding structural information as mutations during reverse transcription, which are then quantified by sequencing. These combined mutations completely abolished binding to the androgen receptor, as demonstrated by RNA electrophoretic mobility shift assay, and reduced melanoma invasion and proliferation [12]. In another example, a single nucleotide change can shift the lincRNA secondary structure. The structural alteration caused by a point mutation in lincRNA NEAT1, predicted by MutaRNA [14] which is based on RNAsnp [15], impacts its binding to miRNAs in colorectal cancer [16]. However, a limited number of studies focus on the mechanisms of lncRNAs’ secondary structures compared to the number of studies reporting novel lncRNAs or identifying differentially expressed lncRNAs (less than 2% of papers on lncRNA and cancers studied RNA secondary structures). One main reason is that the experimental methods used to detect RNA structures are normally expensive and time-consuming. By predicting structural disruption and integrating multi-omics datasets at a genomic scale, we identify cancer-associated SNPs that potentially disrupt lincRNA secondary structures. We then analyze how these disruptions hold the potential to impact protein-lincRNA interactions. These disruptive SNPs are valuable for future analysis that studies the molecular mechanism of lincRNAs in cancer through interacting with other molecules.

Although many biochemical and biophysical methods have been optimized to identify RNA structure at the genome scale, most of them still face bottlenecks [17]. The complete RNA structurome remains largely unknown. The SNPs can cause both local and global RNA structure disruptions. Full-length sequences can be used to predict global structure disruption, but it has risks in the accuracy because the prediction accuracy decreases for long transcripts. Moreover, most SNPs disrupt local region than distant region [11]. A local structural change may block or make available a binding site for other molecules, even though the change is small compared to the structure of the full molecule. The local structural change may therefore highlight biological meaningful changes that will be lost when only considering global changes. Thus, we focus on the local disruption from SNPs to maintain accuracy and obtain the most disruptions of SNPs. However, it will be interesting to study the influence of local changes on distal regions in future studies.

In an early benchmark paper [18], the structural change prediction algorithms were compared on a genome-wide high-resolution RNA structural differences dataset detected by enzymatic probing experiments. This benchmark study found that these three algorithms: remuRNA [19], RNAsnp [15], and SNPfold [20], are best at identifying SNPs that cause structural disruption. A recently published RNA structural differences prediction algorithm named RNAsmc [21] reported better performance than RNAsnp when predicting disruption of SNPs in full-length RNA sequences. To quantify structural differences, RNAsmc calculated a similarity score between wild-type and mutant MFE structures based on comparing structural elements, such as bulge loops, hairpin loops, stems, etc. The similarity score from RNAsmc ranges from 0 to 10, with a score closer to 0 representing higher structural differences. They showed that when using RNAsmc and RNAsnp to identify SNPs that induced global changes, 70% of disruptive SNPs were identified by RNAsmc with the similarity score < 10, and 24% of SNPs were identified by RNAsnp with *P*-value < 0.2 (the recommended threshold of RNAsnp) [21]. However, the similarity score threshold is much looser than the *P*-value cutoff. We inspected the number of RNAsmc identified disruptive SNPs under different cutoffs for similarity scores and found that only 11% of SNPs were identified when the similarity scores were less than 7.5.

Overall, we chose to use RNAsnp because of its accuracy in identifying disruptive SNPs when compared to methods that analyze the global structural changes. Furthermore, RNAsnp predicts the local structural difference, and reports the interval of structurally changed RNA, which is used for the further analyses.

RNAsnp reports the interval with maximum structural changes between wild-type and mutant sequences together with *P*-values. The structural difference is assessed by calculating the Euclidean distance or the correlation coefficient between two structural ensembles. By making use of the pre-computed background tables of structural changes from random sequences, RNAsnp reports *P*-values based on the Gumbel distribution for Euclidean distance or the beta distribution for correlation coefficient. RNAsnp has been applied to predict the structural changes of SNPs in lncRNAs in the lncRNASNP database [22]. This database integrates published databases, including lncRNA expression patterns, diseases associations of lncRNAs and variants, and performs predictions to estimate impacts of variants on lncRNA secondary structures, miRNA-lncRNA interactions, and drug resistance. It provides a comprehensive resource for studying the potential impacts of SNPs on lncRNAs. Compared to the lncRNASNP, our study focuses on the structural change in the local region around the SNP in lincRNA, while lncRNASNP folds the entire lncRNA transcript to predict structural changes. The local structural changes around SNPs are more biologically meaningful than distant structural changes. In addition, lncRNAs can function through structural domains rather than a single structure of the entire transcript [8]. Overall, the local disruption prediction provides prediction accuracy and predict most disruptions caused by SNPs.

The cancer genome includes the accumulation of mutations [23]. Hence, we focus on the structural changes of cancer-related SNPs in lincRNAs and analyze the potential biological impacts of these structural changes at the genomic scale. The data used in this project includes cancer-related SNPs identified in cancer samples by The Cancer Genome Atlas (TCGA) [24] and lincRNAs extracted from GENCODE [25]. RNAsnp was employed to compute the structural effects of SNPs by calculating the Euclidean distance between secondary structure ensembles of wild-type and mutant sequences [15]. We analyzed the biological functions of these structural changes through the following aspects. Firstly, for cancer-associated SNPs and lincRNAs with RNAsnp *P*-values < 0.1, we collected information about their functions through literature curation. Secondly, for all cancer-associated SNPs and lincRNAs, we generated a dataset including the results of structural disruptions of SNPs and the differential expression annotations of lincRNAs in cancers. Thirdly, we compared structurally disrupted regions and RNA binding protein (RBP) targets to identify the influence of structural changes on protein binding for lincRNAs. In addition, we highlighted a set of glioblastoma multiforme related SNP-lincRNA-RBP that is worthy of further analyses to validate whether the structural change impacts the binding of RBP in glioblastoma multiforme.

## Results

### Pipeline for predicting structural disruption

**Fig. 1:**
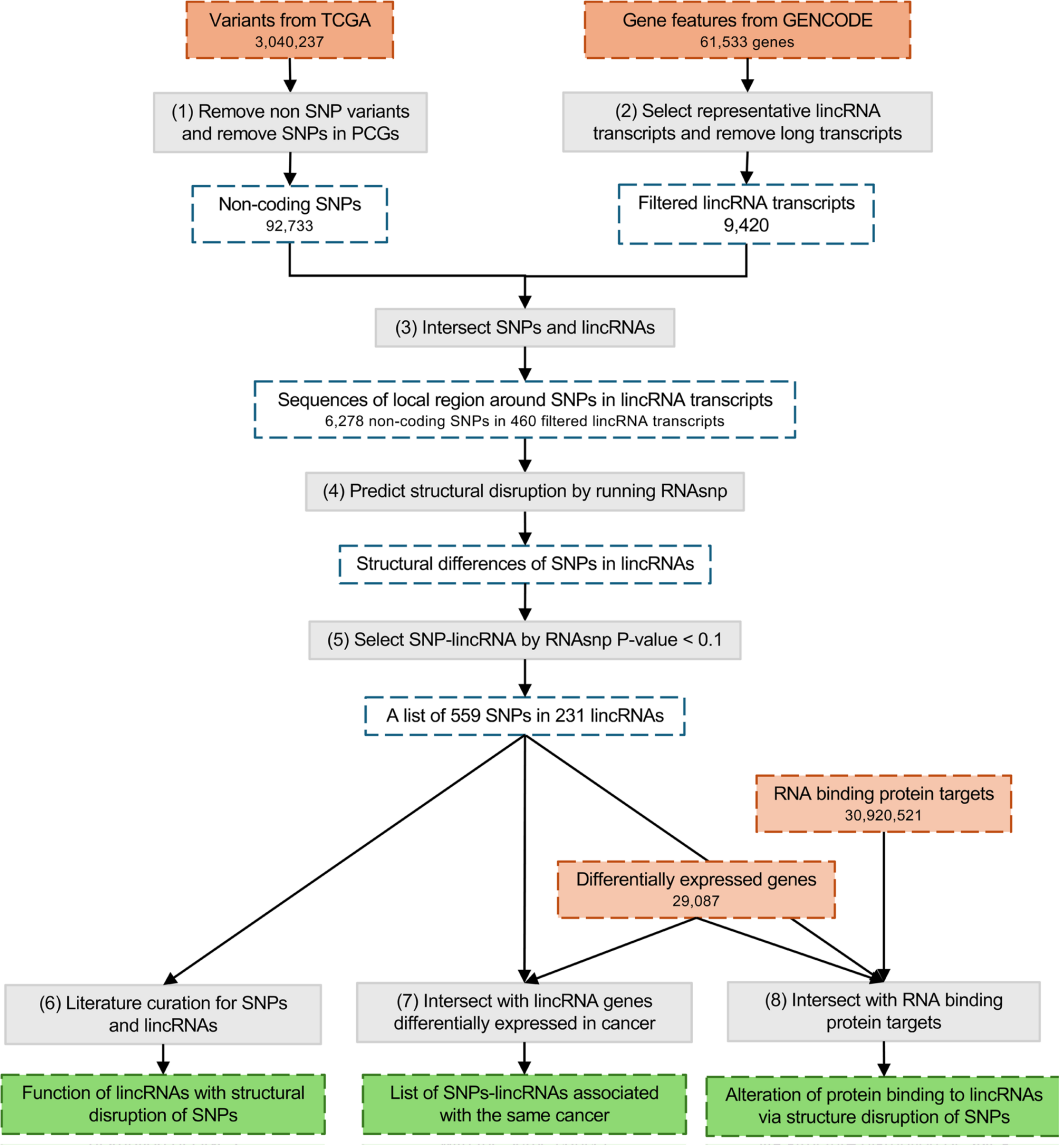
Workflow of structural disruption prediction and functional analyses.(1) SNPs were extracted from cancer-associated variants using the annotation of variant type in TCGA and further filtered by removing SNPs in protein-coding genes. (2) We defined lincRNAs as lncRNAs that do not overlap protein-coding genes. For each lincRNA gene, we selected the Ensembl canonical transcript as the representative transcript to predict structural changes. The representative transcripts were filtered by length, as shown in Fig. 2A. (3) The pair of cancer-associated SNPs and lincRNAs were matched by intersecting their genomic coordinates. (4) The sequences of spliced lincRNA representative transcripts and the transcript position of cancer-associated SNPs were used to run RNAsnp, which computed the structural difference of a local region around the SNP in the spliced lincRNA transcript. (5) A list of cancer-associated SNPs and lincRNAs with RNAsnp *P*-value < 0.1 was selected for downstream analyses. (6) Literature curation in PubMed Central to reveal the function of lincRNAs with structural changes of SNPs. (7) To generate a list of SNPs and lincRNAs associated with the same cancer, the cancer type of the SNP was combined with the cancer types where the lincRNA is differentially expressed according to the lncRNA-cancer databases. (8) The RBP binding sites and structurally disrupted regions in lincRNAs were intersected by their genomic coordinates, generating a dataset of RBPs whose target is in the structurally disrupted region of lincRNAs. Furthermore, differential expression of RBP in cancers were used to identify the SNP-lincRNA-RBP related to the same cancer

**Fig. 2:**
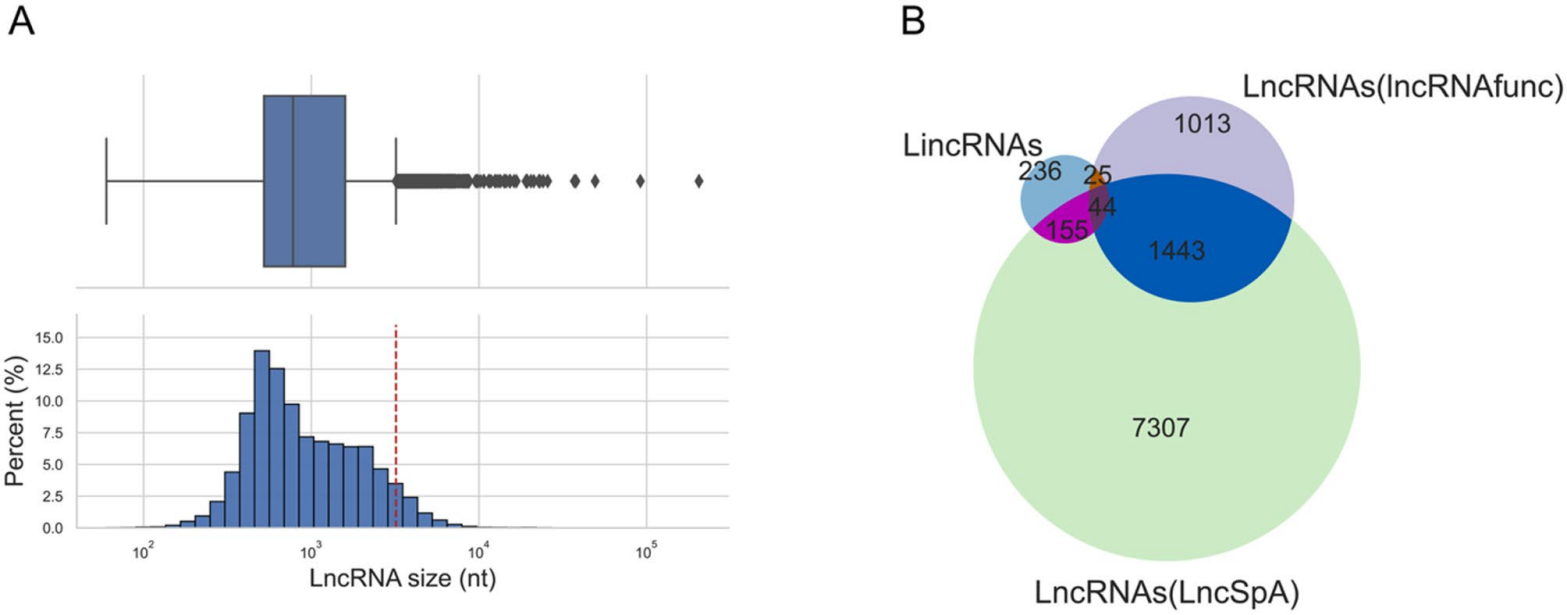
Overview of datasets.** (A)** Boxplot of the length of Ensembl canonical lncRNA transcripts from GENCODE (17,755 transcripts). The box represents the first and third quartiles (Q1 and Q3). The median is shown as a line inside the box. Whiskers extend left (or right) to the smallest (or largest) value greater (or lower) than Q1-1.5*(Q3-Q1) (or Q3 + 1.5*(Q3-Q1)). Values outside whiskers are called outliers because they are numerically distant from the rest of the data (represented by diamonds). Histogram shows the same distribution as the boxplot and the red dashed line represents 3,200 nt, used as a cut-off corresponding to the value of the right whisker in the boxplot. **(B)** Venn diagram of lincRNAs applied structural disruption prediction and lincRNAs with cancer annotation from lncRNAfunc and LncSpA. Among 460 lincRNAs with structural disruption prediction, 199 lincRNAs are annotated in the LncSpA, 69 lincRNAs are annotated in the lncRNAfunc, and 44 lincRNAs are annotated in both databases

As illustrated in the workflow (Fig. 1), we predict local structural changes induced by cancer-associated SNPs in lincRNAs and analyze the biological function of these structural changes. The pipeline is made up of a number of key steps. (i) We collected non-protein-coding cancer-associated SNPs from TCGA [24] by extracting variants annotated as SNPs and removing those in protein-coding genes. (ii) Because there is no lincRNA annotation in GENCODE version 39 [25], we downloaded sequences and genomic feature annotation of lncRNAs to extract the annotation of lincRNAs. For each lncRNA gene, the transcript annotated as canonical by Ensembl was selected [26]. To obtain more confident lncRNAs, we removed transcripts that are considerably longer than the others. The cutoff (< 3,200 nt) was decided by the largest value before outliers as indicated in the box plot (Fig. 2A). The annotation of lincRNAs was extracted by removing lncRNAs that overlap with protein-coding genes (PCGs) without respect to strand. (iii) The list of SNPs that are inside spliced lincRNA transcripts was built by intersecting the genomic coordinates of SNPs and spliced transcripts. The sequences around SNPs in spliced lincRNA transcripts were extracted to be folded into secondary structures to predict the disruption using RNAsnp [15]. (iv) We employed RNAsnp mode 1 to predict structural changes of the region 100 nt up- and down-stream of the SNP (201 nt. in total) in spliced lincRNA transcripts. Thus, SNPs that are closer to either end of the spliced sequence than 100 nt were removed. The size of sequences used for structural disruption prediction is the smallest size in RNAsnp, which is chosen based on the assumption that structural changes farther from the mutated position have less biological importance. To summarize, we applied RNAsnp to predict the structural impacts of 6,278 cancer-associated SNPs in secondary structures of 460 lincRNA transcripts. (v) Using a threshold of RNAsnp *P*-value < 0.1, we extracted 559 cancer-associated SNPs that cause structural disruption in 231 lincRNAs. These SNPs were mostly found in one cancer type (The frequency of cancer types can be found in Supplementary Table S1). (vi) Literature curation for these SNPs and lincRNAs was performed in PubMed Central. (vii) To find the lincRNAs and SNPs associated with the same type of cancer, the SNPs with cancers association were integrated with lincRNAs differentially expressed in cancers, without RNAsnp *P*-value filtering. The annotation was obtained from two databases, lncRNAfunc [27] and LncRNA Spatial Atlas (LncSpA) [28], as described in detail under Methods. (viii) SNPs in lincRNAs were overlapped with RBP binding sites to find the structurally disrupted regions hosting RBP binding sites, regardless of the RNAsnp *P*-value. In addition, the differential expression annotation for both lincRNA and RBP genes was added to further filter our results associated with the same type of cancer. Detailed results are presented later.

To see whether the number of SNPs per lincRNA impacts the selection of lincRNAs hosting structurally disruptive SNPs with RNAsnp *P*-value < 0.1, we compared the number of SNPs in lincRNAs before and after filtering by *P*-values. As shown in Figure S1, the bar plot shows the count of lincRNAs in each bar, which is grouped by the total number of SNPs per lincRNA. The distribution suggests that the lincRNAs hosting SNPs with RNAsnp *P*-value < 0.1 are distributed among the bins in a fashion similar to that of the lincRNAs without *P*-value filtering. Thus, our selection of SNPs and lincRNAs with RNAsnp *P*-value < 0.1 is not biased towards lincRNAs with more SNPs.

### LincRNAs hosting structurally disruptive SNPs

For 559 SNPs and 231 lincRNAs with RNAsnp *P*-value < 0.1, we searched for them in PubMed Central (Details can be found in Methods) and found none of the SNPs have been reported, while 189 lincRNAs have been reported in studies related to cancer. In addition, we manually curated the top 10 lincRNAs ranked by decreasing structural differences and summarized their annotation in Table 1. Overall, 3 lincRNAs have been reported to bind with other molecules, 2 lincRNAs have not been reported, and 5 lincRNAs have been reported as differentially expressed lincRNAs in cancers but lack mechanistic studies.

LINC02913 has been predicted to bind with miRNA hsa-miR-545-3p in papillary thyroid carcinoma. Both LINC02913 and miRNA hsa-miR-545-3p were involved in a competing endogenous RNA network of mRNA nucleolar spindle-associated protein 1 (NUSAP1) in papillary thyroid carcinoma [29].

FAM106A has been reported to be associated with multiple cancers including osteosarcoma, colorectal adenocarcinoma, and breast cancer. In osteosarcoma, FAM106A has been reported to trans-regulate the coding gene AL023806.1 through the transcription factor ZNF169 [30]. The expression of FAM106A has been reported to be down-regulated in colorectal adenocarcinoma cells exposed to the probiotic Lactobacillus acidophilus L-92 [31]. In addition, the enrichment of DNA methylation was observed in the FAM106A promoter region in breast cancer cells treated with anti-cancer components resveratrol. However, the function of enriched methylation is unclear because no change in FAM106A expression was observed [32].

**Fig. 3:**
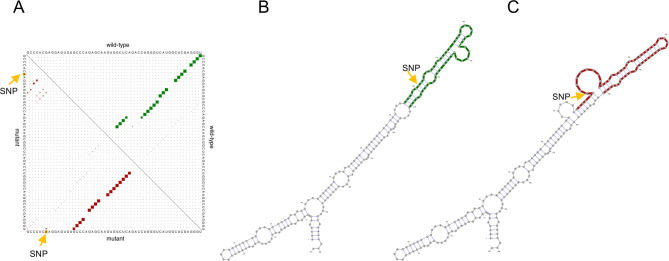
Structural changes in USP27X-DT by SNP at position 685 G > A. The sequence interval with maximum Euclidean distance is from position 679–735 in lincRNA ubiquitin specific peptidase 27 X-linked divergent transcript (USP27X-DT). (**A)** The base pair probabilities matrix of the structural ensemble of the mutant (red) and wild-type (green) sequence interval with maximum Euclidean distance. (**B)** The minimum free energy (MFE) structure of the wild-type sequence. The sequence interval with maximum Euclidean distance is highlighted in green. The bulge at the right side of the stem is at position 722–726 in USP27X-DT. (**C)** The MFE structure of the mutant sequence. The sequence interval with maximum Euclidean distance is highlighted in red. The bulge at the left side of the stem is at position 685–693 in USP27X-DT

Ubiquitin specific peptidase 27 X-linked divergent transcript (USP27X-DT, formerly named USP27X-AS1) was reported to bind with serine and threonine kinase AKT and upregulate AKT signaling in hepatocellular carcinoma [33]. The binding site is at position 1–1,600 where we found a disruption at position 679–735 in USP27X-DT induced by a uterine corpus endometrial carcinoma (UCEC) associated SNP as shown in Fig. 3. The disrupted region includes two changes: more accessibility at position 685–693 (the bulge at the left side of the stem in the mutant structure) and less accessibility at position 722–726 (the bulge at the right side of the stem in the wild-type structure) in mutant USP27X-DT. However, to our knowledge there is no study that has analyzed how lincRNA USP27X-DT functions in UCEC, making it challenging to further infer the function of these structural changes.

**Table 1:** Annotation of top 10 LincRNAs with highest Euclidean distances. The first column shows the name of lincRNAs, followed by the structural disruption levels represented by Euclidean distances and corresponding *P*-values listed in the second and third columns. The last column records the annotation of lincRNAs reported in the literature. Notably, lincRNA LINC02913 has 2 SNPs that are both in the top 10 highest Euclidean distances

LincRNA	Educlidean dist.	*P*-value	Annotation
LINC02915	0.6062	0.0025	Prognostic risk factor of gastric cancer [34]
ENSG00000279151	0.6050	0.0033	Unknown
USP27X-DT	0.5523	0.0095	Bind with protein in hepatocellular carcinoma [33]
ENSG00000278730	0.5446	0.0033	Differentially expressed in treated lymphoma cells [35]
ATP11AUN	0.5299	0.0111	Differentially expressed in lung adenocarcinoma [36]
LINC00207	0.5277	0.0062	Unknown
LINC02913	0.5249^1^ 0.5055^2^	0.0063^1^ 0.0074^2^	Bind with miRNA in papillary thyroid carcinoma [29]
FAM106A	0.5117	0.0054	a. Trans-regulate coding gene in osteosarcoma [30] b. Differentially expressed in treated colorectal adenocarcinoma cells [31] c. Enriched DNA methylation in promoter region in treated breast cancer cells [32]
LINC01641	0.5000	0.0078	Differentially expressed in colorectal cancer [37]
EXOC3-AS1	0.4968	0.0080	Differentially expressed in treated neuroblastoma cells [38]

### LincRNAs differentially expressed in the same cancer where the SNPs are identified

To obtain a list of SNPs and lincRNAs associated with the same cancer types, we combined our results with the annotation of differentially expressed lincRNAs. The annotation is integrated from two databases. One is LncRNA Spatial Atlas (LncSpA), which identified tissue-elevated lncRNAs by comparing the expression in one cancer with the average expression in other cancers [28]. The other is lncRNAfunc, which includes lncRNAs differentially expressed between tumor and matched normal samples [27]. Among 460 lincRNAs for which we performed structural disruption prediction, 224 lincRNAs are annotated in at least one database. 199 are annotated in the LncSpA database and 69 are annotated in the lncRNAfunc database. There are 44 lincRNAs annotated in both databases **(**Fig. 2B**).** The full list, including structural disruptions of SNPs-lincRNAs and the associated cancers, can be found in Supplementary Table S2. Based on the differential expression annotation of lincRNAs in cancer, 41 out of 224 lincRNAs are differently expressed in the same cancer where the corresponding SNPs are identified. The cancer frequency of lincRNAs (all lincRNAs and lincRNAs with SNPs) can be found in Figure S2.

We highlight 8 lincRNAs that host 9 structurally disruptive SNPs with RNAsnp *P*-value < 0.1 and are differentially expressed in the same cancers where SNPs are identified **(**Table 2**).** More than half of the SNPs (5 out of 9) are from UCEC samples, which is consistent with the cancer frequency of SNPs in lincRNA transcripts as shown in Figure S3. There are two UCEC-associated SNPs with RNAsnp *P*-value < 0.1 causing structural disruption in the same lincRNA rhophilin Rho GTPase binding protein 1-antisense RNA 1 (RHPN1-AS1). RHPN1-AS1 is an oncogene in many cancers [39] and its molecular mechanisms have been studied in several cancers, including endometrial cancer where RHPN1-AS1 participates in the ERK/MAPK signaling pathway [40].

**Table 2:** List of LincRNAs associated with the same cancers where SNPs are identified. The table lists eight lincRNAs associated with the same cancers where SNPs are identified and RNAsnp *P*-value of SNPs < 0.1. The first column shows the gene name of lincRNAs and the second column lists cancers where lincRNAs are differentially expressed, together with the name of lncRNA-cancer databases (lncRNAfunc [16] or LncSpA [17]). Cancer names in bold indicate that a SNP(s) in this lincRNA is also identified in this cancer. The pair of abbreviations and full names of cancers together with SNPs’ position can be found in Supplementary Table S2

LincRNAs	Associated cancers
ENSG00000212978	KICH; **UCEC** (lncRNAfunc)
LINC01139	KICH; HNSC; **UCEC**; LUSC (lncRNAfunc) | OV; THYM (LncSpA)
LINC01124	**COAD**; LIHC; READ (lncRNAfunc) | LIHC (LncSpA)
FAM41C	**OV**; THYM (LncSpA)
MIR3142HG	**ESCA**; COAD; READ (lncRNAfunc) | SKCM; UVM (LncSpA)
RHPN1-AS1	**UCEC**; LUSC; BRCA; LUAD (lncRNAfunc)
SNHG25	**GBM**; KIRC; BLCA; KIRP; UCEC; PRAD; LUSC; BRCA; LUAD; COAD; LIHC; READ (lncRNAfunc) | OV (LncSpA)
LINC00868	TGCT; **UCEC** (LncSpA)

### Cancer-associated SNPs impact protein binding with LincRNAs by disrupting RNA structure

It is widely known that lincRNAs can function through interacting with RBPs [41]. One important mechanism of RBP binding is recognizing and binding to RNA secondary structure [42]. To analyze the impacts of structural disruption on RBP binding, we compared the structurally disrupted region in lincRNAs with RBP binding sites that are identified by crosslinking and immunoprecipitation (CLIP) based experiments. The annotation of RBP binding sites was downloaded from the POSTAR website [43]. In total, more than 30 million binding sites of 220 RBPs identified from eight CLIP associated methods. The counts of RBP targets from different methods are shown in Figure S4. The cancer frequency of RBPs is shown in Figure S5.

By intersecting RBP binding sites and the structurally disrupted candidate regions in lincRNAs, we found 706 (324) RBP binding sites for 109 (79) RBPs in structurally disrupted regions predicted by RNAsnp with *P*-value < 0.1 (0.05) (Table 3).

**Fig. 4:**
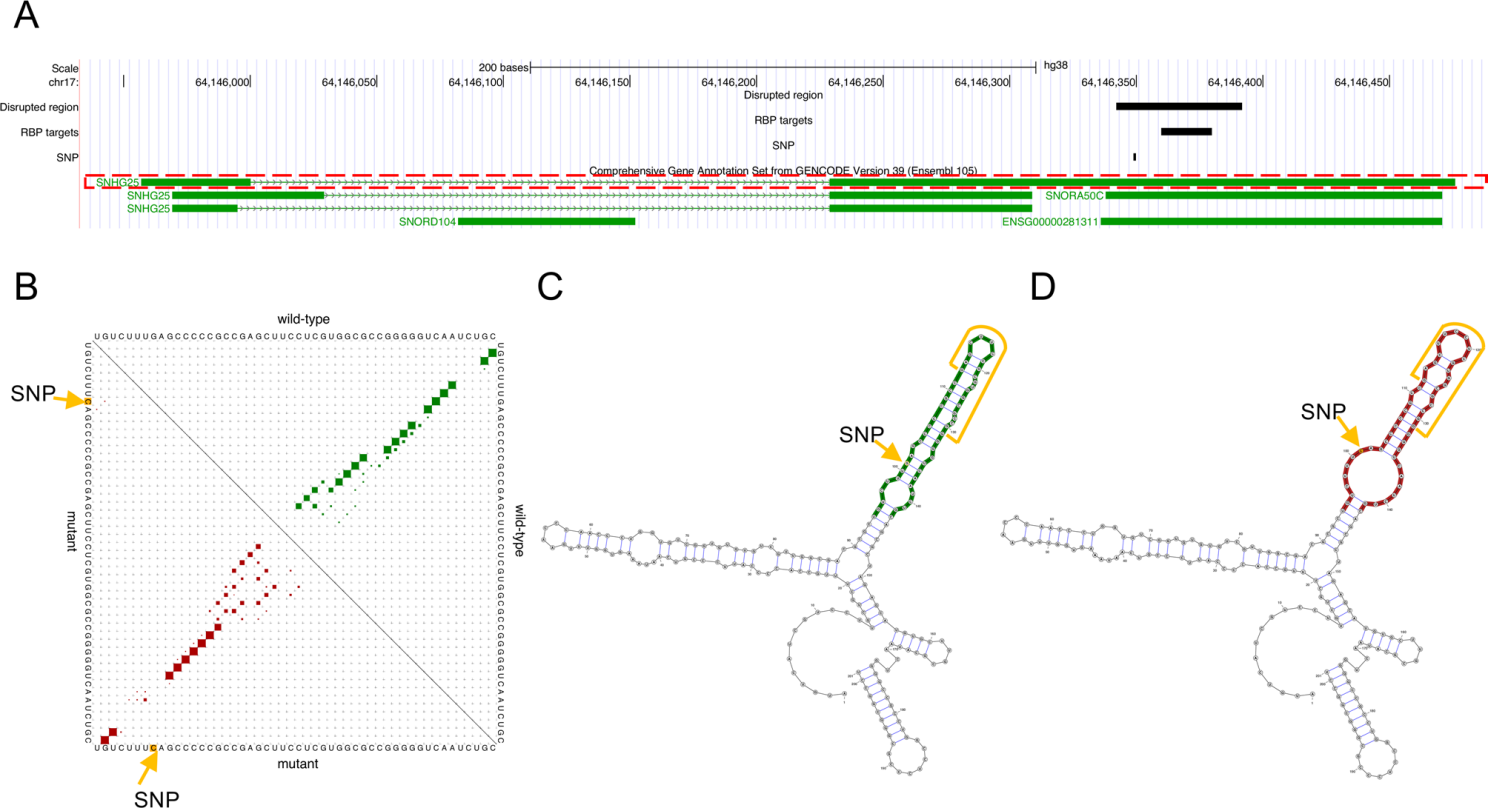
SNP at 164 G > C in SNHG25 disrupts the secondary structure and impacts protein binding. The sequence interval with maximum Euclidean distance is from position 157–206 in lincRNA SNHG25. (**A**) Genomic position of SNHG25, the SNP, the structurally disrupted region, and the targets of RBP IGF2BP2. The main transcript of SNHG25 used in the analysis is circled with the red dashed line. (**B**) The base pair probabilities matrix of the structural ensemble of mutant (red) and wild-type (green) sequence intervals with maximum Euclidean distance. (**C**) The minimum free energy (MFE) structure of the wild-type sequence and the sequence interval with maximum Euclidean distance are highlighted in green. RBP binding site is indicated by yellow lines in **C** and **D**. (**D**) The MFE structures of the mutant sequence and the interval with maximum Euclidean distance are in red

Among all structurally disrupted regions that host RBP binding sites, we highlight an example in the lincRNA small nucleolar RNA host gene 25 (SNHG25). This predicted structural disruption (RNAsnp *P*-value = 0.08) is based on a glioblastoma multiforme related SNP at position 164 in SNHG25, and the disrupted region overlaps 27 RBP binding sites from 19 RBPs. Insulin like growth factor 2 mRNA binding protein 2 (IGF2BP2) has five targets in SNHG25 identified in the glioblastoma multiforme cell line (MGG8) and one target inside the disrupted region. The other targets can be found in Figure S6. As illustrated in Fig. 4, the SNP induced more unpaired bases in loops in the mutant secondary structure of SNHG25. These induced loop regions partly overlap the IGF2BP2 binding site suggesting higher RNA accessibility for protein binding (highlighted by yellow lines in Fig. 4C and D). In addition, both the lincRNA SNHG25 and the RBP IGF2BP2 are up-regulated in glioblastoma multiforme according to the annotation from lncRNAfunc [27]. Although SNHG25 shows potential as biomarkers in several cancers, limited studies have focused on glioblastoma multiforme. Its first mechanism was reported in 2022 [44], and no study has been done regarding the protein binding of SNHG25 in glioblastoma multiforme. However, SNHG25 was proven to function through interaction with protein dyskerin pseudouridine synthase 1 (DKC1) in neuroblastoma cells [45]. We infer that SNHG25 is likely to function by binding with IGF2BP2 in glioblastoma multiforme.

**Table 3:** Number of molecules regarding structurally disrupted region hosting RBP targets. The full list of structurally disrupted regions and intersected RBP binding sites can be found in Supplementary Table S3. Results with other P-value cut-off can be obtained from Supplementary Table S3. RBP is the number of proteins with their RBP targets overlapping SNP induced structurally disrupted regions inside lincRNAs

RNAsnp *P*-value	SNP	LincRNA	RBP	RBP targets
< 0.05	43	30	79	324
< 0.1	97	50	109	706

## Discussion

In this study, we predicted the local structural changes from cancer-associated SNPs on the secondary structure of lincRNAs. We employed RNAsnp to calculate Euclidean distances between RNA secondary structural ensembles of the local region around SNPs in wild-type and mutant lincRNAs. We investigated the biological function of the lincRNAs with structural disruptions from cancer-associated SNPs. For the local disrupted region in lincRNAs, we estimated the impacts of protein binding in the interval with maximum structural differences. As a result, we found a SNP induced structural change in lincRNA SNHG25 increases the binding probability of N^6^-methyladenosine (m6A) methylation reader protein IGF2BP2. This finding provides valuable insights into the currently unclear mechanism of m6A methylation recognition.

To our knowledge, no study has analyzed the function of SNPs from TCGA whole exome sequencing data in non-coding regions because these SNPs were expected only in protein-coding exons. However, a study found a significant number of high-quality SNPs outside the target regions in various whole exome sequencing datasets [46]. We indeed identified more than 6,000 SNPs in spliced lincRNA transcripts from TCGA exome sequencing data. Our research demonstrates the existence of SNPs in intergenic regions from TCGA whole exome sequencing datasets and functionally analyzes these previously ignored SNPs.

Considering that RNAsnp was executed 6,480 times for the 6,278 SNPs, *P*-values from RNAsnp should in principle be adjusted for multiple testing correction to control the false discovery rate. However, we used RNAsnp *P*-values without multiple testing correction for the following two reasons: One, the individual cases shown in this paper eliminate false positive structural changes because we analyzed the base pair matrix of structural ensembles and minimal free energy structures before analyzing their biological importance. Two, the distribution of *P*-values (Figure S7A**)** includes a peak near *P*-value = 1, which is not applicable for multiple test correction methods that require a flat distribution of *P*-values. In addition, the distribution of *P*-values does not indicate the error in the RNAsnp computation algorithm but naturally reflects the distribution of Euclidean distances (Figure S7B**)**. Moreover, the RNAsnp *P*-values are with low resolution, which results in a discrete distribution that is not suitable for general multiple testing procedures. RNAsnp can be improved by applying suitable multiple testing procedures to discretely distributed *P*-values in the future.

By inspection in the literature database using the keywords lincRNA name and “structure”, we found no literature that reported the function of RNA secondary structure in the same region as the predicted structurally disrupted regions in spliced lincRNA transcripts in our results. This is not unexpected because less than 2% of the literature studied lncRNA and cancer also analyzed RNA secondary structure and cancer. The lack of functional study of lincRNAs is evident when exploring the known mechanism of lincRNAs with the top 10 highest structural changes. Only 3 out of these 10 lincRNAs have been reported to function through binding to miRNA (LINC02913 binds to hsa-miR-545-3p) and protein (FAM106A binds to ZNF169 and USP27X-DT binds to AKT). Moreover, USP27X-DT is the only example detected by biomolecular experiments, while the interactions of the other two lincRNAs are from prediction. However, some lincRNAs related to the same cancer as the SNP might have been missed in this study because we only obtained differential expression data for lincRNAs from two databases, which is far from a complete differential expression annotation. The list of SNP-lincRNAs related to the same cancer and with RNAsnp *P*-value < 0.1 is shown in Table 2. All SNP-lincRNAs related to the same cancer type are presented in Supplementary Table S2.

By comparing genomic regions with structural changes to the genomic regions targeted by RBPs, we identified that 50 out of 231 lincRNAs have structurally disrupted regions with RNAsnp *P*-value < 0.1, where RBPs bind. These RBPs include both canonical RBPs that prefer to bind specific RNA sequences and non-canonical RBPs, e.g., fused in sarcoma (FUS) which has been identified to recognize and bind RNA by both RNA sequence motif and structure motif [42]. FUS binds with the interval with maximum structural differences in four lincRNAs: ENSG00000270066, ENSG00000261069, URB1-AS1, and SNHG32. However, none of the MFE RNA secondary structures of these intervals match the target recognition mode of FUS. One possible reason is that the FUS target recognition mode was analyzed in solution, structure and the binding mode is more complex in the cell.

We identified a set of SNP-lincRNA-RBP (164:G >C - SNHG25 – IGF2BP2) that are all highly associated with glioblastoma multiforme and potentially involved in the regulatory network related to m6A methylation. The SNP was identified from a glioblastoma multiforme patient. We predicted that it disrupts a local secondary structure of lincRNA SNHG25 that is up-regulated in glioblastoma multiforme. A binding site of RBP IGF2BP2 was observed in this disrupted region in a glioblastoma cell line, and the IGF2BP2 gene is also up-regulated in glioblastoma multiforme. The differential expression of SNHG25 and IGF2BP2 suggests their biological function in glioblastoma multiforme. In addition, IGF2BP2 is a m6A methylation reader and is associated with prognosis in glioblastoma patients [47]. Although the snoRNAs are in the same genomic region as SNHG25 (Fig. 4A), they are unrelated to m6A methylation because snoRNAs guide pseudouridylation and 2′-O-methylation. The structural change, resulting in fewer base pairs in the mutant SNHG25 structure, favors the binding between SNHG25 and IGF2BP2 because IGF2BP2 is a single-stranded RBP [48]. By comparing the m6A sequence motif (GGACU) identified in glioblastoma cells with the sequence of the IGF2BP2 binding site in SNHG25, we found the binding site is unlikely to be m6A-methylated. This m6A motif was identified from methylated RNA immunoprecipitation sequencing experiments without bias toward RNA types [47]. IGF2BP family proteins have been reported to read m6A signals through K-homology 3–4 domains, but the K-homology 3–4 peptides alone are not sufficient to recognize m6A [48]. It is possible that the binding between IGF2BP2 and SNHG25 is needed to recognize the m6A signal. The interaction between SNHG25 and IGF2BP2 can also be involved in other functions independent of the m6A mechanism. The binding between IGF2BP2 and RNA can protect both RNA and IGF2BP2 from degradation or facilitate the degradation [49]. Considering the overexpression of both SNHG25 and IGF2BP2 in our example, the interaction may protect both SNHG25 and IGF2BP2 from degradation. Hence, more analyses, such as other m6A modified RNAs and SNHG25 co-expression analysis, are warranted to study whether the binding between IGF2BP2 and SNHG25 facilitates the m6A modified RNA recognition or not. The binding between SNHG25 and IGF2BP2 may also facilitate the interaction with other proteins that bind to SNHG25 or IGF2BP2. To our knowledge, there is no protein has been reported physically interact with SNHG25 or IGF2BP2. However, there are several proteins that are in the same complex with IGF2BP2. It will be valuable to screen all proteins that bind with SNHG25 in glioblastoma to study whether SNHG25 interacts with IGF2BP2 and promotes the formation of a complex including SNHG25, IGF2BP2 and other proteins. Future biomolecular experiments are warranted to study the precise position in IGF2BP2 that SNHG25 (both wild-type and mutant) binds to and the impacts of this binding on recognizing m^6^A-modified mRNAs in glioblastoma multiforme.

The computational analyses reported here are an exploratory study of the disruption from cancer-related SNPs on the secondary structure of lincRNAs. Our results associate cancer-related SNPs to disruption of the local secondary structures in lincRNAs that are differentially expressed in the same cancer type where SNPs are identified. These SNPs, lincRNAs, and the structural changes are worthy of focus when investigating molecular mechanisms in cancers. We furthermore associate the structurally disrupted regions where RBPs bind, which can be used in follow-up studies for further experimental validation of how SNPs alter protein binding of lincRNA by disrupting their secondary structures. To comprehensively study the biological meaning of structural changes in lincRNAs, future analyses should focus on the impacts of structural changes on lincRNAs’ binding to RNAs and DNAs. In addition, the workflow presented in this study can be applied to investigate other groups of lncRNAs and SNPs. For example, SNPs related to certain phenotypes or other lncRNAs. There are only RNA sequencing data available for 6% of the patients (675 out of 10188 patients) that host SNPs. Therefore no differential expression analysis between the wild-type and mutant lincRNAs has been performed.

We explored the enrichment/depletion of Gene Ontology (GO) terms for lincRNAs with structural disruption by running gene set enrichment analysis (GSEA). GSEA was performed for lincRNAs by introducing neighboring PCGs and variable screen windows were set to find neighboring PCGs. However, no significant (FDR q-value of GSEA < 0.1) enriched/depleted GO terms were identified. It is worthwhile to explore the enriched/depleted GO terms or pathways for lincRNAs with structural disruption in the future by using co-expressed PCGs of lincRNAs. As our understanding continues to grow, focusing on lincRNAs may be too strict, given that the human genome is not linearly arranged with a clear boundary between coding and non-coding regions [8].

## Methods

### LncRNA and SNP related data processing

The lncRNA and protein coding gene annotations were downloaded from GENCODE v39 [25]. The link of the annotation file can be found in Availability of data and materials. For lncRNA genes with multiple transcripts only the transcript annotated as canonical by Ensembl [26] was used for structural prediction. To obtain more confident lncRNAs, we removed lncRNA transcripts that are considerably longer than others based on the threshold (3,200 nt) indicated in the distribution of lncRNA length (Fig. 2A). We made a sub-dataset for lincRNAs by removing lncRNAs that overlap PCGs. When checking the overlap between lncRNA and PCGs, we consider the start and end coordinates of all transcripts from the same gene (for both lncRNA gene and PCG). The list of lincRNAs was extracted by running the BEDtools intersect command [50] to find lncRNAs that have no overlap with PCGs (-v option) without respect to strand. The overlap was based on direct genomic coordinates overlap, without extending the genomic regions of PCGs. We extracted SNPs from TCGA simple nucleotide variation data according to the information on variant type. The simple nucleotide variation data was downloaded using the gdc-client from the Genomic Data Commons (GDC) Data Portal on February 9, 2021. The command for running gdc-client and the file (Su) used to download variation data were described in Availability of data and materials. The list of SNPs located inside lincRNA transcripts was made based on their genomic coordinates by running the BEDtools intersect command. In total, we collected 6,278 cancer SNPs identified from 10,188 patients and these SNPs are in 460 lincRNAs (Figure S8).

### Cancer-associated genes datasets

We obtained the association between gene expression and cancers from two databases. The links of both datasets can be found in Availability of data and materials. One is lncRNAfunc including both protein-coding genes and lncRNA genes that are differentially expressed between tumor and matched normal samples [27]. The DESeq2 pipeline [51] was used in lncRNAfunc to identify differentially expressed genes based on criteria of adjusted *P*-value < 0.05 and |log2FC| >1. The second database is LncSpA which identified tissue-elevated lncRNAs by comparing the expression in one cancer with the average expression in other cancers [28] and selected 5-fold higher expression levels in one tissue compared with all other tissues. We downloaded differentially expressed lncRNA genes from both lncRNAfunc and LncSpA. We also downloaded differentially expressed protein-coding genes from lncRNAfunc to analyze proteins that bind to the structurally disrupted region in lincRNA.

### RNA binding protein target sites dataset

The annotation of RBP binding sites was downloaded from POSTAR3 under the CLIPdb module [43]. The link for requesting the dataset can be found in Availability of data and materials. POSTAR3 includes a large collection of RBP binding sites detected from various CLIP experiments. In total, there are 30,920,521 binding sites of 220 RBPs identified by 8 methods. In total, three methods were used to process data from CLIP experiments. PureCLIP [52] was for data from iCLAP, urea-iCLIP, 4sU-iCLIP, eCLIP, iCLIP. CLIPper [53] was used for HITS-CLIP data. MiClip [54] was used for PAR-CLIP data. All three methods were run with default parameters, which use 0.05 as the cutoff to filter out significant binding signals. A small fraction of RBP binding sites (356,838 out of 30,920,521) detected by PIP-seq were removed because PIP-seq is designed to pull down all RNAs binding to protein without information of RBP [55]. The distribution of RBP binding sites from different methods is shown in Figure S5.

### Prediction of structural disruption from SNP in LincRNA

RNAsnp was used to do the RNA folding and distance calculation, with options: Mode 1 which is designed for short sequences and window size 100 which sets the screening window to 100 nt upstream and downstream to the SNP (201 nt total). The default values were used for other parameters including: 0.01 was used as the minimum cut-off for the base pair probabilities and 50 was used as the minimum length of the sequence interval. Because the window size is 100 nt, we removed SNPs that are closer to either end of the spliced sequence than 100 nt. We ran the command line RNAsnp v1.2 to predict the structural changes of SNPs in all lincRNAs by running with command lines under Linux. The RNAsnp web server [56]was used to generate MFE structure figures and base pair probability matrix for wild-type and mutant sequences of interest.

### Manual curation using literature databases

We systematically searched the literature for the SNPs with RNAsnp *P*-value < 0.1 and the corresponding lincRNAs in the PubMed Central (PMC) database on September 3, 2023. Both pairs of lincRNA-SNP and individual lincRNAs or SNPs were searched for. To search for literature reporting the same lincRNA-SNP pair as in our project, the gene name of lincRNA and the genomic coordinates of SNPs are connected by “AND”. Then all lincRNA-SNP pairs are connected by OR” to form a term to search. For SNPs recorded in dbSNP, we also searched for their dbSNP ID. To find literature for lincRNAs, we used the term that consists of lincRNA names connected by the logical operator “OR” to search. To find literature for SNPs, we used the term that consists of genomic positions of SNPs or dbSNP IDs if both coordinates and dbSNP IDs were available they were combined with “OR”. For lincRNAs listed in Table 1, we also searched their gene names in PubMed and Google Scholar and manually checked the literature to obtain comprehensive results. To avoid missing literature because of changes to the lincRNA names, we checked the information for the lincRNAs in Table 1 in GeneCards [57], because the literature presented in here covers publications related to all versions of the gene name. We searched the lincRNA gene name from Table 1 one by one and checked the literature listed under the publication.

## Supplementary Information

Supplementary Material 1

Supplementary Material 2

Supplementary Material 3

Supplementary Material 4

Supplementary Material 5

## Data Availability

Data used in the present study are publicly available. TCGA data was downloaded through the following command: gdc-client download -m Supplementary_table_4.xlsx. The gdc-client is available here: https://gdc.cancer.gov/access-data/gdc-data-transfer-tool. The links to download other data are listed below: Gene annotation: https://ftp.ebi.ac.uk/pub/databases/gencode/Gencode_human/release_39/gencode.v39.annotation.gff3.gz. LncRNA differential expression (LncSPA): https://bio-bigdata.hrbmu.edu.cn/LncSpA/download/TCGA_classification.txt?x=17&y=14. LncRNA differential expression (lncRNAfunc): https://ccsm.uth.edu/lncRNAfunc/download_tables/1-Differntial_expression_lncRNA_in_TCGA/AllCancerType_TumorVSNormal_difflncRNAgene.txt. PCG differential expression: https://ccsm.uth.edu/lncRNAfunc/download_tables/1-Differntial_expression_lncRNA_in_TCGA/AllCancerType_TumorVSNormal_diffPGgene.txt. The path of downloading CLiP dataset was obtained upon request through the link: https://wj.qq.com/s2/10477617/4f84/v.
