# Supplementary material for: Associating cancer-related RNA structure disrupting SNPs in LincRNAs to function
Source: BMC Genomics. 2025 Dec 12;26:1100. doi: 10.1186/s12864-025-12226-0 (PMC12701586; doi:10.1186/s12864-025-12226-0)
Supplement: Supplementary file 1 — Supplementary Material 1 [file 12864_2025_12226_MOESM1_ESM.docx]

**Associating cancer-related RNA structure disrupting SNPs in lincRNAs to function**

Xueer Han^1^, Christian Anthon^1^, Adrian Sven Geissler^1^, Radhakrishnan Sabarinathan^2^, Stefan Ernst Seemann^1^, Jakob Hull Havgaard^1*^, Jan Gorodkin^1*^

1. Department of Veterinary and Animal Sciences, Center for non-coding RNA in echnology and Health, University of Copenhagen, Denmark

2. National Centre for Biological Sciences, Tata Institute of Fundamental Research, Bengaluru, India

*Correspondence: gorodkin@rth.dk; hull@rth.dk

**Supplementary Information**


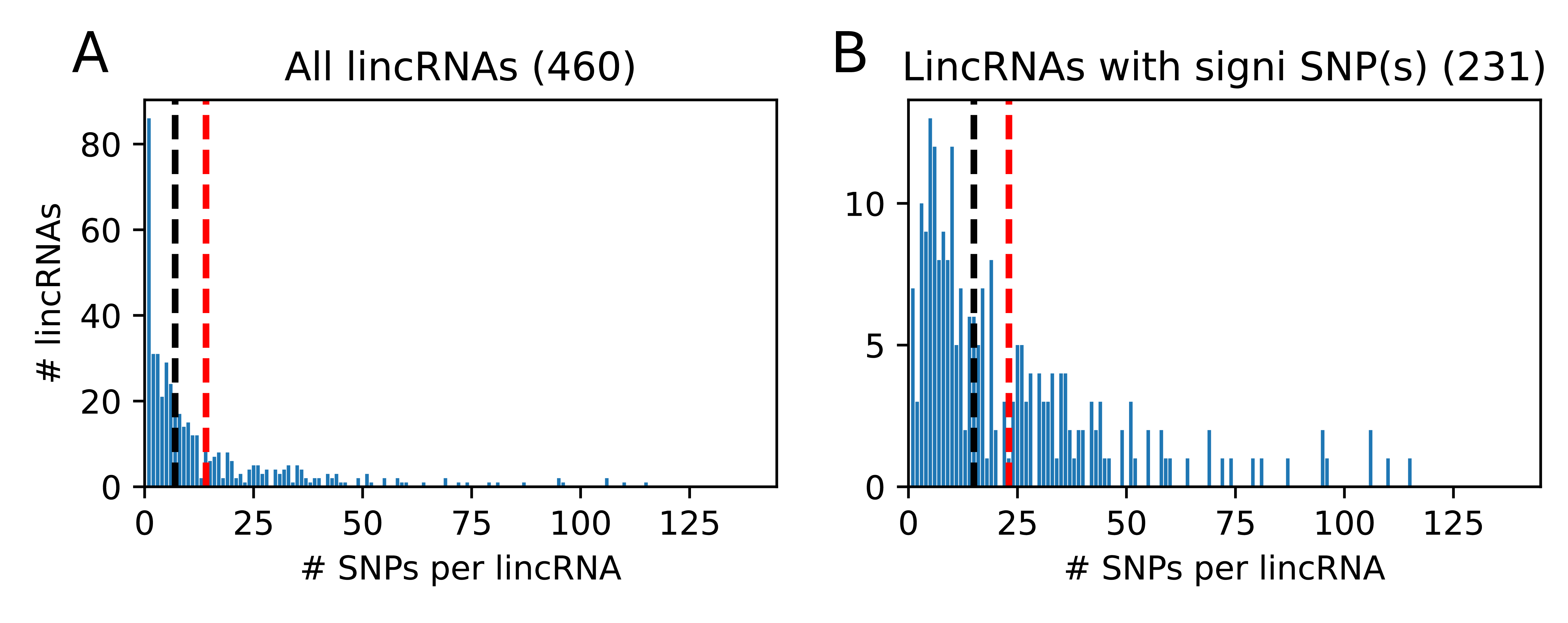


**Figure S1. Distribution of the number of SNPs per lincRNA.** Bar plots show the different numbers (#) of SNPs in lincRNAs (x-axis) and the counts (#) of lincRNAs (y-axis). The number of lincRNAs is indicated in the title of each plot. The median is indicated by the black dashed line, and the average number is in the red dashed line. **A**. includes all lincRNAs used in predicting structural changes. **B**. includes only the lincRNAs that host at least one significant SNP. The number of SNPs, as indicated on the x-axis, represents all SNPs in the lincRNA regardless of significance.


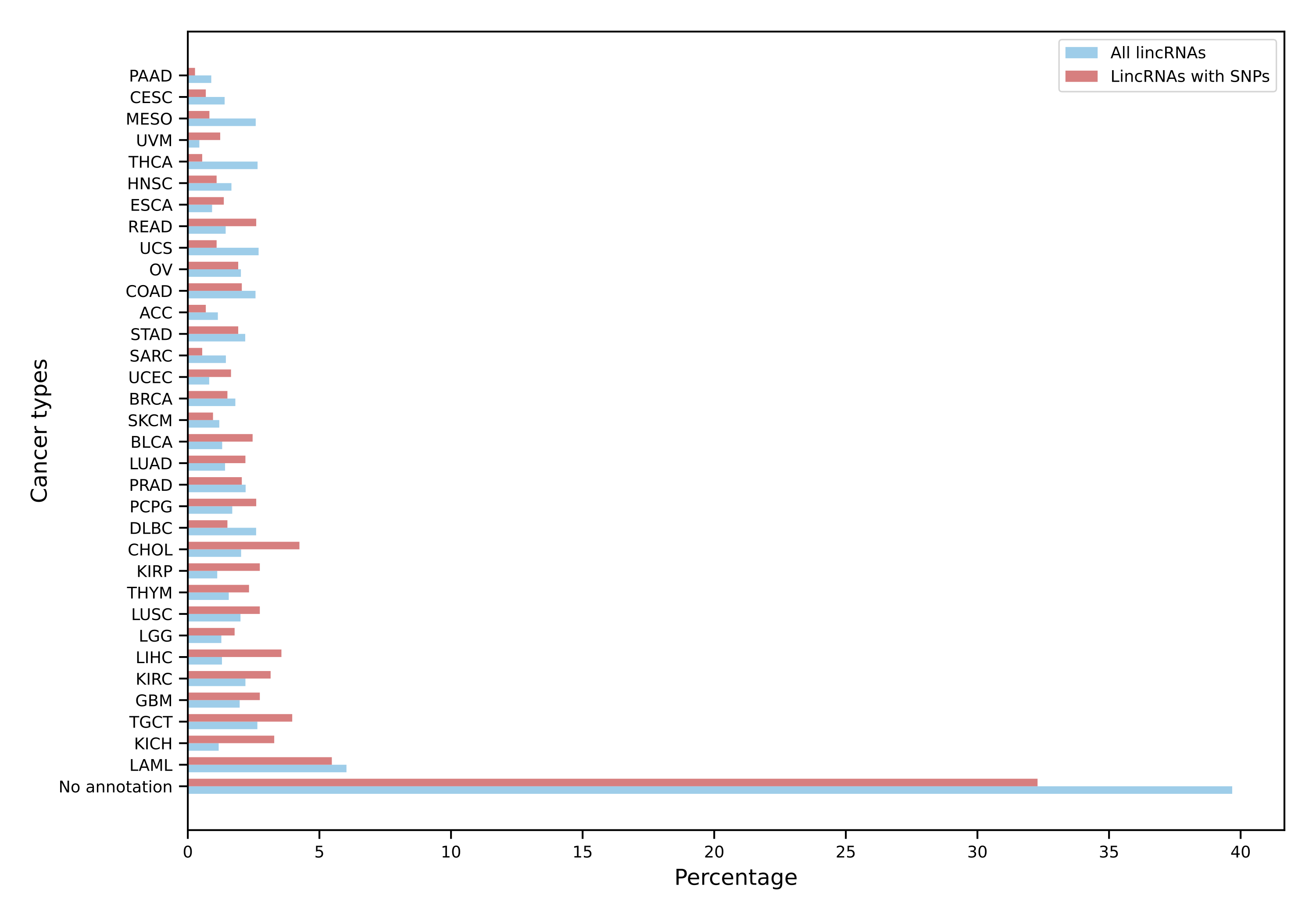


**Figure S2. Frequency of lincRNAs.** The percentage of all 9,427 lincRNAs and 460 lincRNAs hosting SNPs used in the project in each cancer.


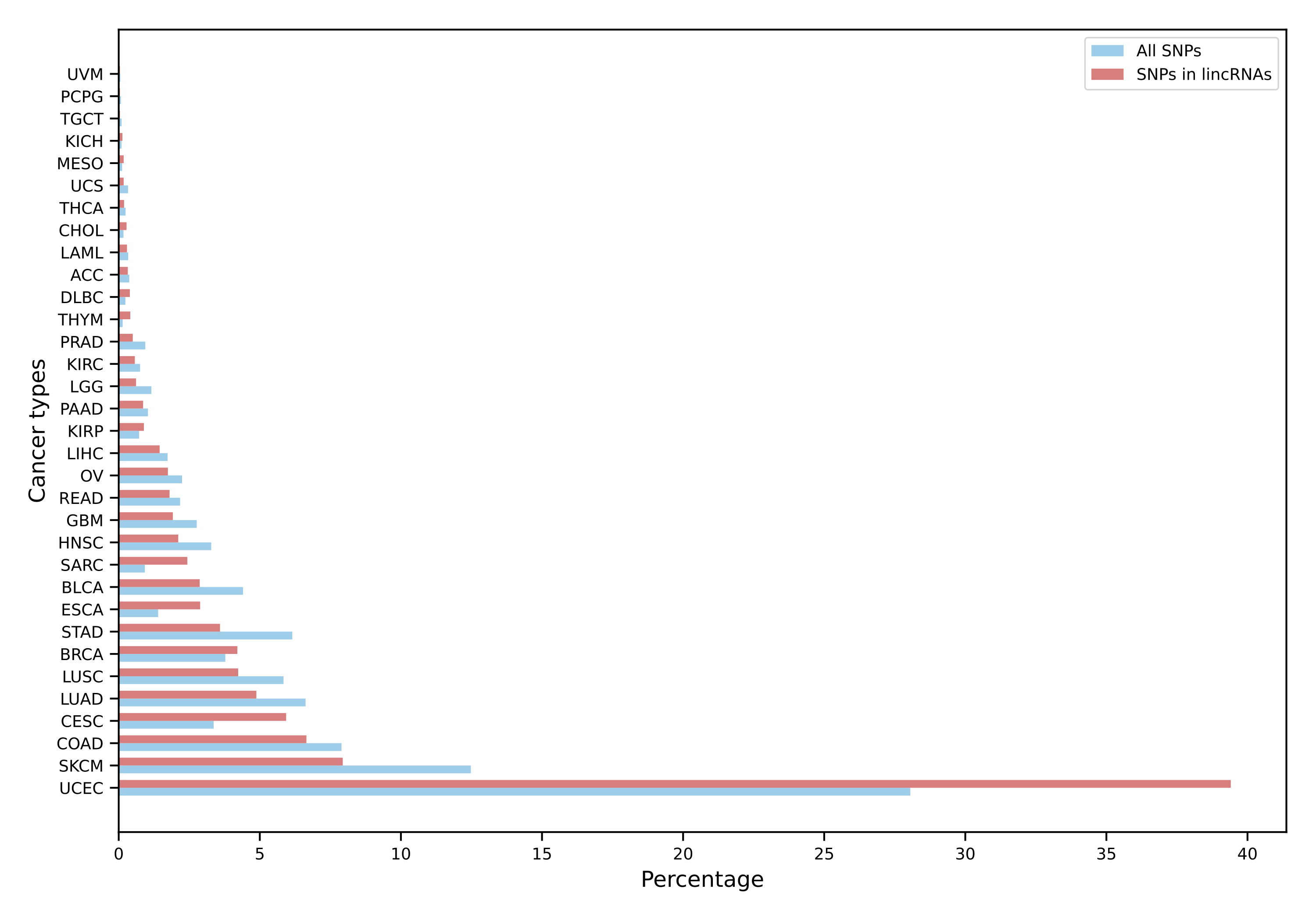


**Figure S3. Frequency of SNPs.** The percentage of 6,278 SNPs that are located in lincRNAs and all 2,854,811 SNPs from TCGA is shown in a bar plot for each cancer type (Y-axis).


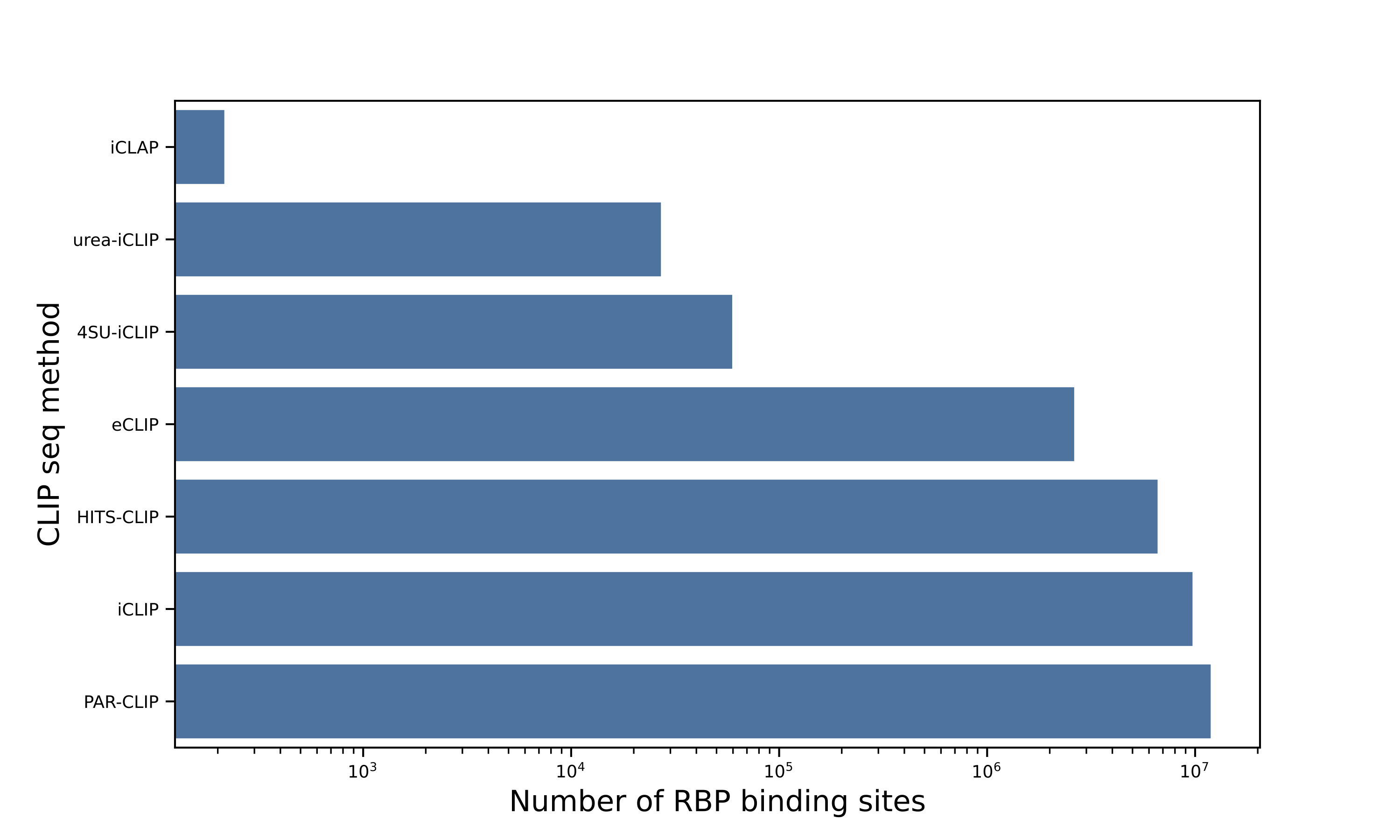


**Figure S4. Distribution of counts of RBP binding sites from different experimental methods.** The experimental methods used in identifying RBP binding sites include individual-nucleotide resolution crosslinking affinity purification (iCLAP), urea-iCLIP, 4-thiouridine (4SU) -CLIP, enhanced CLIP (eCLIP), high-throughput sequencing of RNA isolated by CLIP (HITS-CLIP), individual-nucleotide resolution CLIP (iCLIP), and photoactivatable ribonucleoside-enhanced CLIP (PAR-CLIP).


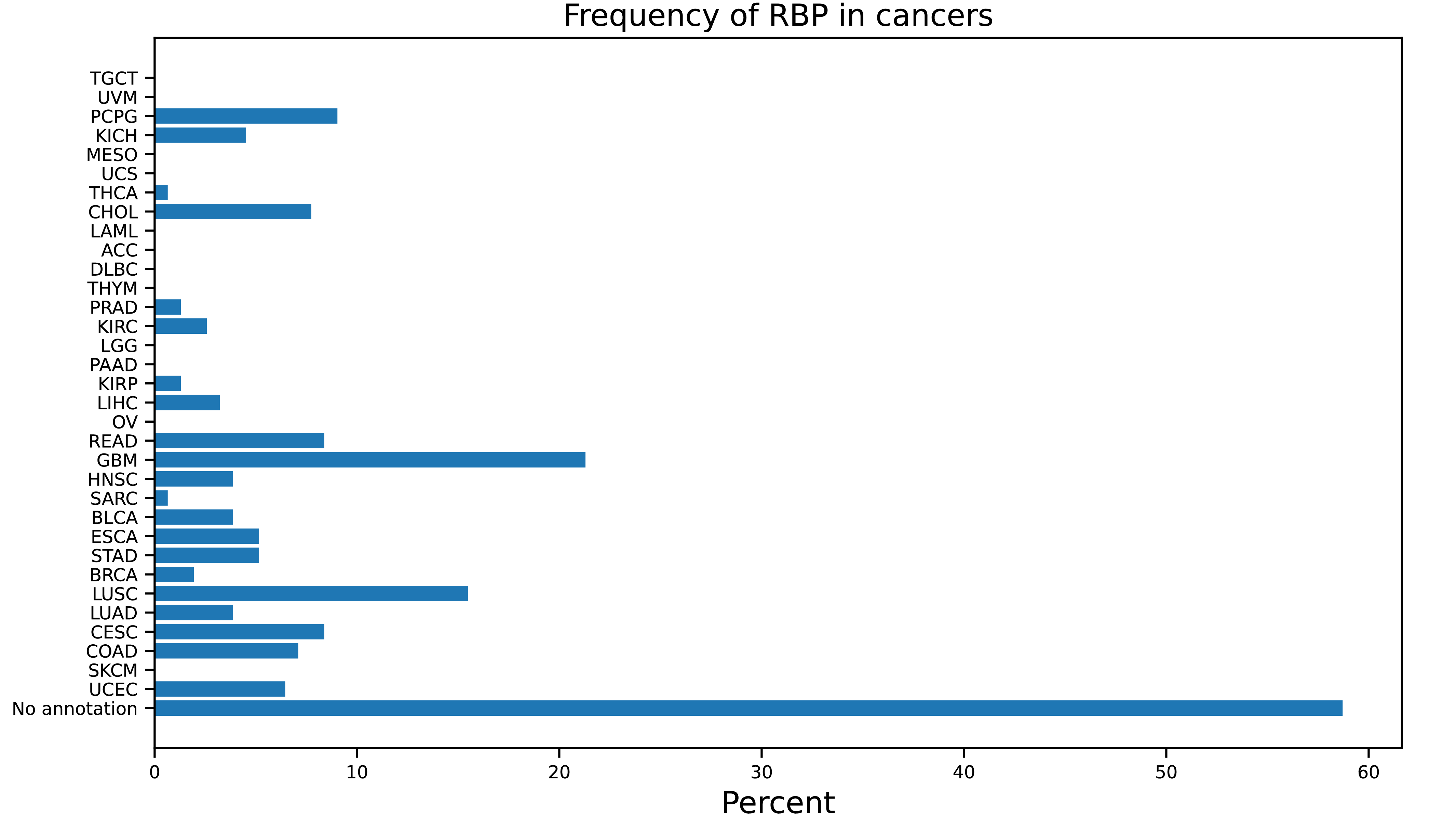


**Figure S5. Frequency of RBPs in each cancer.** The percentage of 155 RNA binding proteins (RBP) used in the project in each cancer (y-axis).


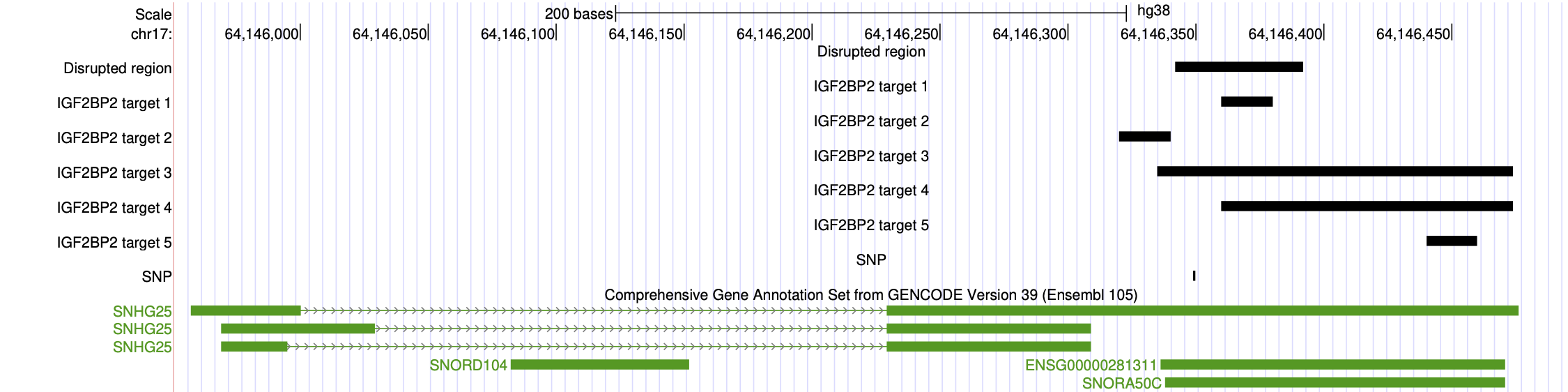


**Figure S6. The binding site of IGF2BP2 in SNHG25.** The disrupted region represents the sequence interval with maximum Euclidean distance is from position 157 – 206 in lincRNA SNHG25. Genomic position of the SNP, the structurally disrupted region, and five targets of RBP IGF2BP2 are shown in black tracks.

**
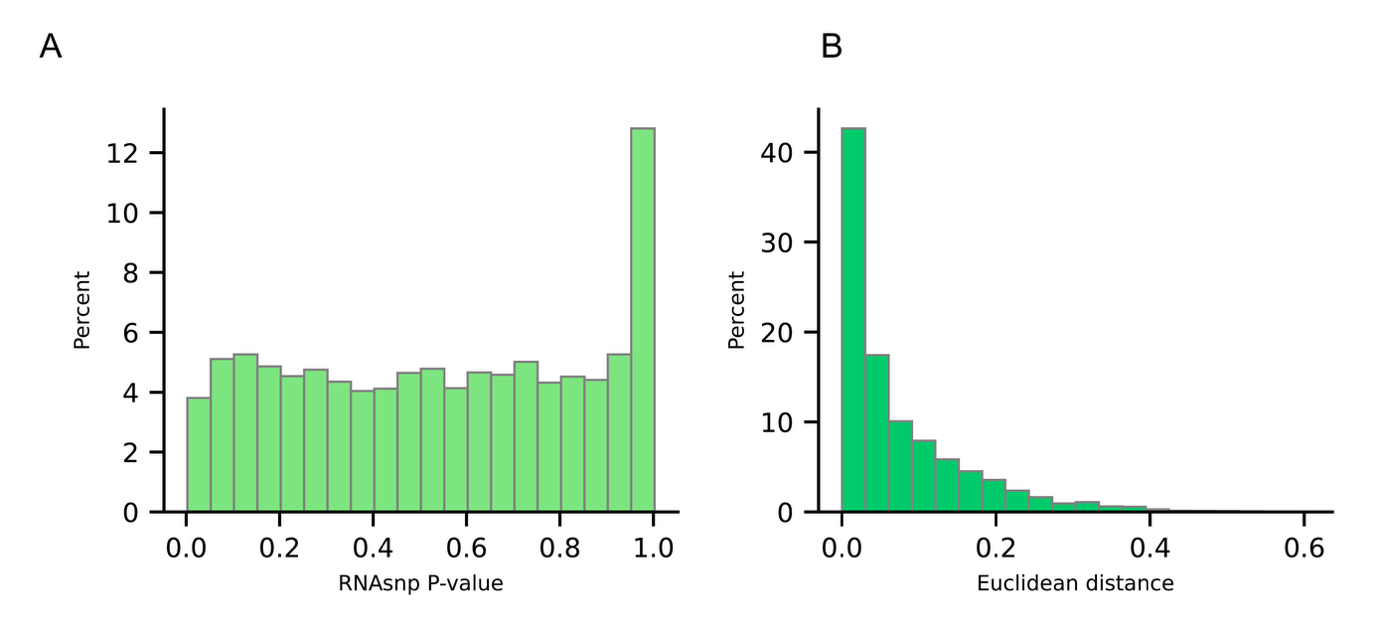
**

**Figure S7. Distribution of structural disruption of all SNPs in lincRNAs. (A)** Distribution of RNAsnp [1] *P*-values of Euclidean distances and the width of the bar is 0.05. **(B)** Distribution of Euclidean distance between secondary structure ensembles of mutant and wild-type sequences. The sequence is the interval with maximum Euclidean distance from sequence 100 nt down- and up-stream of SNP in spliced lincRNA transcripts.

**
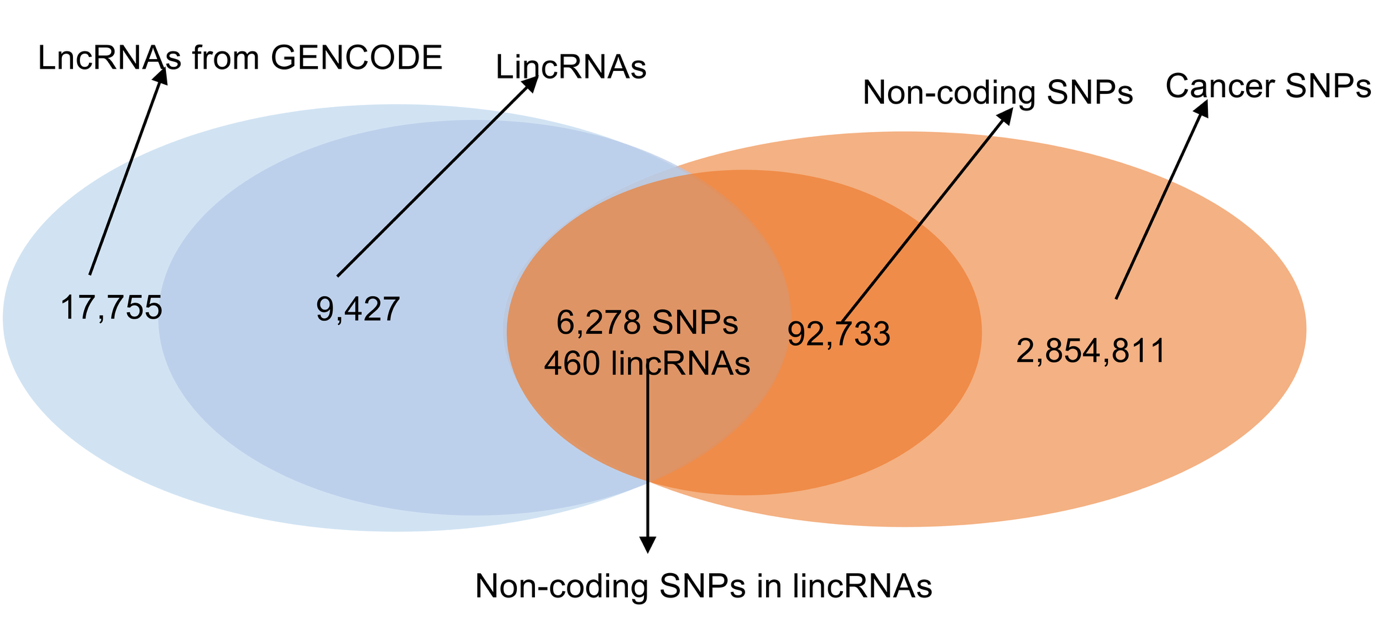
**

**Figure S8. Numbers of lncRNAs and SNPs.** The number of lncRNAs and SNPs before and after processing. We extracted lincRNAs from lncRNAs by removing lncRNAs that overlap PCGs. For cancer SNPs, we used non-coding SNPs by removing the SNPs in PCGs. The number of SNPs is calculated by counting the genomic coordinates of SNPs and the number of prediction results is more than the number of SNPs because SNPs can have multiple alternative alleles.

**Table S1. Frequency of cancer types for 559 SNPs with RNAsnp P-value < 0.1.** The cancer name and counts are listed in this table. The cancers combined by semicolon (highlighted in red) represent same SNP identified in multiple cancers.

**Table S2. Dataset of structural prediction results for all SNPs.** The 1^st^ column shows the transcript ID of lincRNAs and the transcript position of SNP. The 2^nd^ column shows the genomic coordinates, reference allele, alternative allele, and strand of SNP. The 3^rd^ and 4^th^ columns show Euclidean distance and P-value from RNAsnp of SNP. The 5^th^ column shows genomic coordinates and strands of the disrupted region. The 6^th^ column lists cancer type where SNP is identified. The 7^th^ to 10^th^ columns show the gene name, gene ID, transcript length of lincRNAs, and the total number of SNPs in this lincRNA. The 11^th^ column represents the cancer type where lincRNA is differentially expressed together with the name of lncRNA-cancer databases (lncRNAfunc [2] and LncSpA [3]). The last column records the shared cancer types between SNPs and lincRNAs.

**Table S3. Dataset of all structurally disrupted regions hosting RBP targets.** The 1^st^ column lists the transcript ID of lincRNA and the transcript position of SNP. The 2^nd^ column shows the P-value from RNAsnp. The 3^rd^ column shows the genomic coordinates, reference allele, alternative allele, and strand of SNP. The 4^th^ to 6^th^ columns list the gene name, gene ID, and transcript length of lincRNA. The 7^th^ column lists the genomic coordinates of the disrupted region. The 8^th^ column displays the genomic coordinates of RNA binding protein (RBP) targets. The gene name of RBP is shown in the 9^th^ column. The 10^th^ column lists the sample and experiment method used to identify RBP targets. The cancer where the RBP gene (or lincRNA gene) is differentially expressed is listed in the 11^th^ column (or 13^th^ column) together with gene-cancer databases (lncRNAfunc[2] and LncSpA [3]). The 12^th^ column shows the cancer where SNP is identified.

**Table S4. The variation data IDs.** The study IDs and relevant information of variation data from TCGA, which were used to download the dataset through the gdc-client tools.

**References:**

1. Sabarinathan, R., et al., *RNAsnp: efficient detection of local RNA secondary structure changes induced by SNPs.* Hum Mutat, 2013. **34**(4): p. 546-56.

2. Yang, M., et al., *lncRNAfunc: a knowledgebase of lncRNA function in human cancer.* Nucleic Acids Res, 2022. **50**(D1): p. D1295-d1306.

3. Lv, D., et al., *LncSpA: LncRNA Spatial Atlas of Expression across Normal and Cancer Tissues.* Cancer Res, 2020. **80**(10): p. 2067-2071.
